# Silliman’s Chemistry

**Published:** 1847-10

**Authors:** 


### SILLIMAN'S CHEMISTRY.

Benj. Silliman, Jr., Professor, in Yale College, of Science as Applied to the Arts, is the author of a neat duodecimo volume on Chemistry, of nearly five hundred pages; and of the numerous treatises on this subject designed especially for the use of colleges and schools, we do not remember a single one superior to it in merit. It presents a clear, satisfactory view of the first principles of the science, just such as the student requires at the beginning, neither so minute in detail as to weary and perplex, nor so condensed as to be dry and unintelligible. We shall take great pleasure in recommending it to the students in the University of Louisville as a text-book, and are persuaded that a better work could not be placed in the hands of those about entering upon the study of chemistry. The illustrations, amounting to more than two hundred, will aid the student materially in comprehending the apparatus, processes and experiments described in the text. We cannot doubt that the work will be extensively introduced into our colleges and schools. Its publishers are Loomis and Peck, Philadelphia.

More than once we have expressed the belief that nearly all the elementary works on chemistry embraced too much, their very copiousness proving an embarrassment to the young student. This impression, we find, is gaining ground, and in the preparation of the work before us it is evident that the judicious author has borne this point in mind.

